# Evolution of indirect reciprocity under emotion expression

**DOI:** 10.1038/s41598-025-89588-8

**Published:** 2025-03-17

**Authors:** Henrique Correia da Fonseca, Celso M. de Melo, Kazunori Terada, Jonathan Gratch, Ana S. Paiva, Francisco C. Santos

**Affiliations:** 1 INESC-ID and Instituto Superior Técnico, Universidade de Lisboa, IST-Taguspark, 2744-016 Porto Salvo, Portugal; 2 DEVCOM U.S. Army Research Laboratory, Playa Vista, CA 90094 USA; 3 Gifu University, 1-1 Yanagido, Gifu, 501-1193 Japan; 4 University of Southern California, 12015 E Waterfront Dr, Los Angeles, CA 90094 USA

**Keywords:** Emotion expression, Reputation, Indirect reciprocity, Cooperation, Evolutionary game theory, Evolutionary theory, Evolution, Social evolution

## Abstract

Do emotion expressions impact the evolution of cooperation? Indirect Reciprocity offers a solution to the cooperation dilemma with prior work focusing on the role of social norms in propagating others’ reputations and contributing to evolutionarily stable cooperation. Recent experimental studies, however, show that emotion expressions shape pro-social behaviour, communicate one’s intentions to others, and serve an error-correcting function; yet, the role of emotion signals in the evolution of cooperation remains unexplored. We present the first model of IR based on evolutionary game theory that exposes how emotion expressions positively influence the evolution of cooperation, particularly in scenarios of frequent errors. Our findings provide evolutionary support for the existence of emotion-based social norms, which help foster cooperation among unrelated individuals.

Explaining cooperative behaviour remains a cross-disciplinary challenge^1–3^: despite being ubiquitous in human society, its associated cost deems it mathematically and evolutionarily irrational. Evolutionary Game Theory (EGT) formalizes explanations from across multiple disciplines to answer this so-called cooperation dilemma^2,4–7^, such as that of reciprocal altruism^8–12^. One form of reciprocal altruism, Indirect Reciprocity (IR), presents an elegant cooperation-enabling mechanism between unrelated individuals: cooperation pays because it confers the image—or reputation—of a valuable community member to the cooperator^11,13,14^. Several social norms—sets of rules that determine the moral value of actions to attribute reputations to individuals—have been highlighted as successful promoters of cooperation^10,15–19^. Despite such extensive research on (i) social norms and reputations; (ii) the growing experimental evidence that emotion expression shapes decision-making and cooperation^20–22^; and (iii) considerable evolutionary literature on how the experience and imitation of emotion might affect cooperation and social-welfare of structured populations^23–26^, no theoretical work to date considers the role of emotions expressed by a cooperator or a defector in the evolutionary success of reputation-based cooperation^27^. As suggested by Robert Frank, modelling moral emotions in models of rational choice is essential to provide realistic insights from theoretical models of cooperation, as emotions can be used as a reliable signal to identify cooperators^28,29^.

The study of emotion expressions through an evolutionary lens started with Charles Darwin in his book, *The Expression of the Emotions in Man and Animals*^30^, which posed the hypothesis that facial expressions are behavioural adaptations that evolved as a result of natural selection to serve communicative functions. Since then, there has been a growing body of experimental evidence showing that emotional expressions serve important social functions^22,31–36^ and constitute cooperative signalling systems^37^. Accordingly, a recent behavioural study showed how emotional expressions play a central role in reputation-based cooperation by shaping reputation-assessment rules—or social norms^20^. The study demonstrated how participants used others’ facial expressions to determine the moral value of their actions; highlighted an emotion-based social norm, arising empirically from the participants’ moral assessments; and concluded that emotional expressions are particularly helpful in the disambiguation of complex social situations and serve an intention-signalling function.

In light of these recent empirical findings, we propose a computational model based on EGT that searches for evolutionary support for Darwin’s hypothesis. This model studies the interplay between emotional expressions and strategies under different levels of noise-inducing errors. Such errors are known to undermine tit-for-tat strategies and simpler social norms such as Image Scoring (IS), where cooperation often hangs at the mercy of a first wrong move, typically triggered by errors^10,11,17,38–40^. We consider a finite population of *z* individuals, who interact in the classical economic game known as the prisoner’s dilemma, extensively used throughout literature as an abstraction of social interactions in which the individual pursuit of self-interest conflicts with collective outcomes. As expected in models of IR^10,11,19,40,41^, agents’ actions in the prisoner’s dilemma are prescribed by two pure strategies—*Always Cooperate* (AllC) and *Always Defect* (AllD)—and two strategies conditional on their counterpart’s reputations—*Discriminate* (Disc): cooperate with Good individuals and defect otherwise; and *paradoxical Discriminate* (pDisc): cooperate with Bad individuals and defect otherwise. Agents also express a set of emotions based on their Emotional Profile (EP): (virtual) players with cooperative EPs express joy upon mutual cooperation and regret upon exploiting the other player (defecting while being the target of cooperation), whereas players with competitive EPs express regret upon mutual cooperation and joy upon exploiting the other. Both types of players express anger upon being exploited and neutral emotion expressions on mutual defection. In our computational model, EPs are simplified to these two sets of expressed emotions—emulating combinations of verbal and non-verbal behaviour such as facial expressions, body posture, prosody, speech, *etc*. A fixed and universal emotion-based social norm then may assign actions a moral value regarding their valence, the participating individual’s reputation and expressed emotions. Following relevant literature on IR models^19,38,42^, the dissemination of such reputations is assumed to be perfect and immediate, through mechanisms such as gossip^43,44^.

Following the seminal model of Ohtsuki and Iwasa^41^, one generation consists of *z* Monte Carlo time steps, allowing all individuals to revise their strategy on average once. During each generation, an individual’s evolutionary traits (strategy and emotional profile) can change either through random mutation, with probability $\mu$ or through a Monte Carlo social learning step. In this step, the individual plays *z* games with randomly chosen opponents in a well-mixed population, after which the individual compares their fitness to that of another randomly selected agent, who has also played *z* games. The individual then stochastically adopts the other agent’s traits, based on the Fermi update rule (see Supplementary Information (SI)). Each complete simulation is comprised of a large number of generations and yields a cooperation index $\eta$, the average number of interactions that lead to donations as a fraction of the total number of interactions. We assume three types of errors throughout the simulation: action execution, reputation assessment and reputation assignment errors. Execution errors ($\varepsilon$) simulate the inability of individuals to act in the way that their strategy dictates^45^, preventing intended actions from occurring with probability $\varepsilon$. Reputation assessment errors (occurring with probability $\alpha$) lead individuals to perceive the wrong reputation of their counterparts, affecting the action prescribed by their strategy^38^. Finally, reputation assignment errors ($\chi$) model situations in which the observer may fail to attribute an accurate reputation to the donor, due to myopic assessment of the reputation of the potential receiver or due to a misinterpretation of the action employed^38,41^.

**Table 1:**
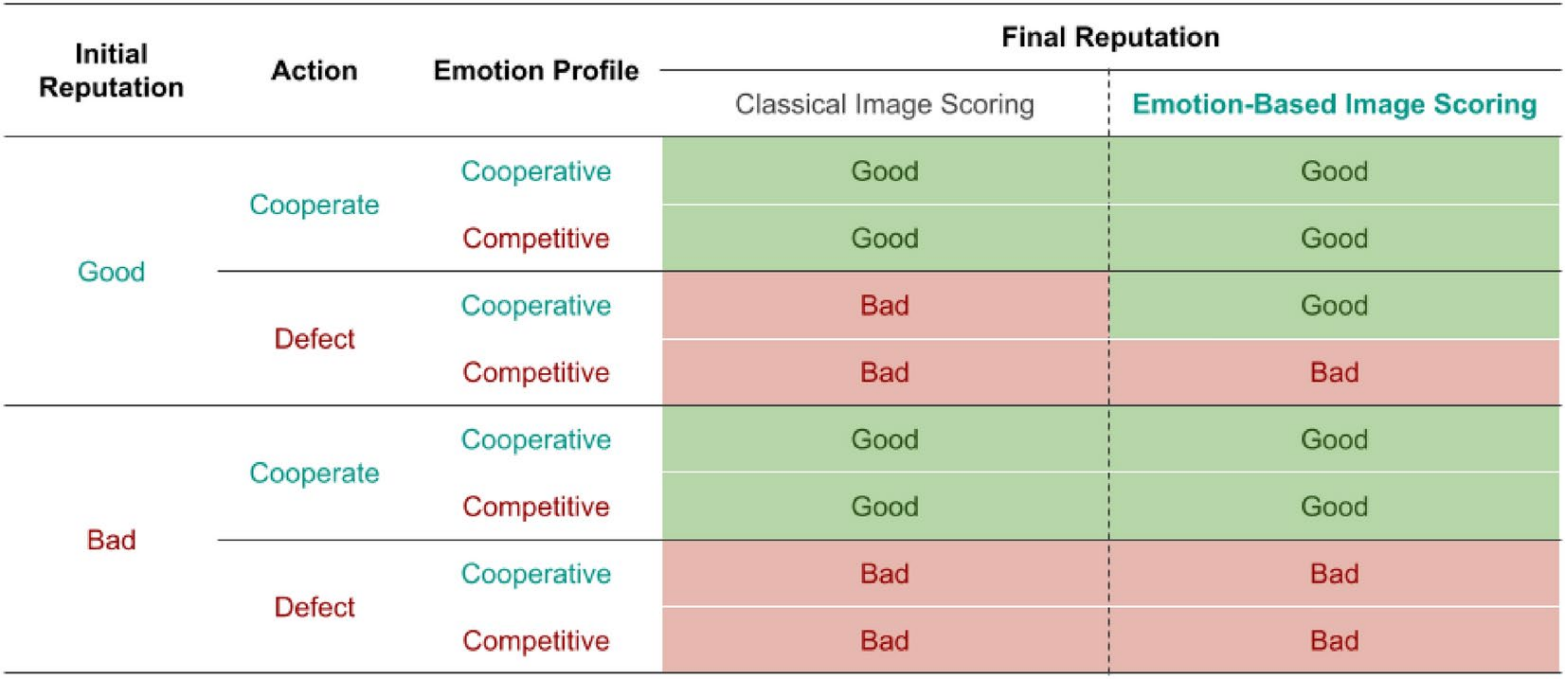
Comparison of the normal version and an emotion-based version of the social norm.

Here we present the studied social norms regarding the donor’s initial reputation $r_{donor}$, the donor’s action $s_{donor}$ and expressed emotion set $EP_{donor}$, and the consequent final reputation $r'_{donor}$. On the left-hand side, we depict a conversion of the classical norm Image Scoring to our structure mindful of expressed emotions, constituting a baseline of comparison with emotion-based norms. Note this norm’s indifference towards emotion: cooperative acts lead to good reputations whereas defective ones yield bad reputations. On the right-hand side, we show the studied emotion-based social norm, based on previous empirical work. Notice how it presents a small but significant distinction from the baseline norm, regarding how a good, but defecting donor should be judged when presenting cooperative emotional expressions.

Following results from empirical studies^20^, we study an emotion-based social norm focusing on the donor’s action, reputation and expressed emotion, as can be seen in Table 1. Notably, this norm structure differs from common literature on IR, even when omitting emotion, as social norms commonly consider the reputations of the recipients of an action, instead of donors^11,17–19,46,47^. Regardless, we focus on these donor-focused social norms as (i) this norm structure was studied by the authors in the original work on emotion-based norms, on which our model is based, and (ii) the main social norm studied in this work, deriving from the original study, constitutes a variation of the simple norm Image Scoring (IS), which cares little about the point of view: according to this simple assessment rule, good and bad reputations come from cooperation and defection, respectively^10^.

We also introduce a parameter controlling the probability that the emotion-based social norm is used to judge an action, in opposition to the classic base social norm. This parameter—which we denote as $\gamma$—emulates different issues that may arise when emotional expressions are necessary to make such judgements: the magnitude and intensity of emotional expressions can be regulated to levels where recognising them becomes non-trivial^22^, these can be omitted or ignored, or even perceived as not genuine^48,49^. Thus, in our model, actions are judged by an emotion-based social norm with probability $\gamma$, represented by the vector $r' = (r_{gcn},r_{gcm},r_{gdn}, r_{gdm},r_{bcn},r_{bcm},r_{bdn},r_{bdm})$, where *c*, *d*, *g*, *b*, *n*, *m*, *r* stand for cooperation, defection, good, bad, cooperative EP, competitive EP and reputation respectively (for the sake of differentiating through a unique symbol, the letter *n*—as in “nice”—stands for the cooperative EP and the competitive EP is represented by the letter *m*—as in “mean”, with no necessary semantic correlation to the original profile denominations, having the single purpose of differentiating them through a unique symbol). With the complementary probability $1-\gamma$, the moral evaluation defaults to a baseline “emotion-free” social norm, resembled by the tuple $r' = (r_{gc}, r_{gd}, r_{bc}, r_{bd})$. Thus, for low $\gamma$ values, emotional expressions are often disregarded by the social norm, which defaults to donor action and reputation, whereas for high $\gamma$ values, emotional expressions are more often considered in the moral evaluation step. With the described model, we start by exploring the cooperation dynamics under an emotion-based social norm, with IS as a baseline, by adapting an empirically found emotion-based social norm^36^ to our model with binary reputations and EPs. Given our binary simplification, we obtain the following emotion-based norm: $r'=({G}, {G}, {G}, {B}, {G}, {G}, {B}, {B})$, henceforth refered to as Emotion-Based Image Scoring (EBIS), as can be seen and compared to IS in Table 1. The specific methodology used to obtain this social norm and comparisons with other possible emotion-based norms can be seen in the Supplementary Information.

## Results

**Fig. 1:**
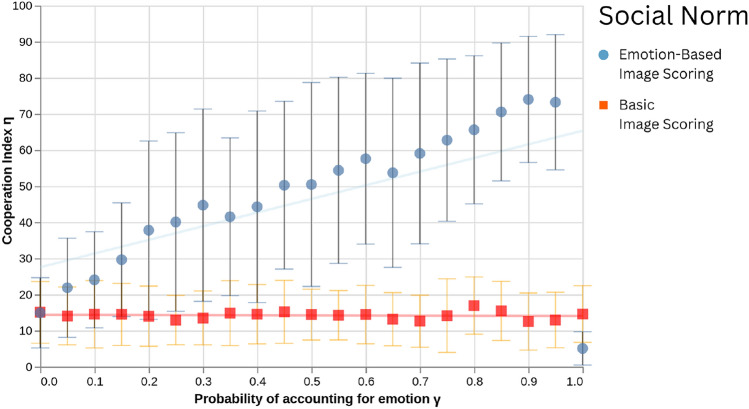
Average cooperation ratio ($\eta$) (and respective standard deviation) promoted by the emotion-based image scoring norm (blue circles) and the baseline image scoring (orange squares), for the different probabilities of accounting for emotion ($\gamma$), when all errors—execution, assessment and assignment—are present ($\alpha =\chi =\varepsilon =0.002$). While the (low) performance of Image Scoring remains consistent with the literature, by looking at the performance of emotion-based image scoring we obtain clear benefits by using emotional expressions as part of the moral evaluation process—higher $\gamma$ values lead to higher cooperation levels under the emotion-based norm, while having no impact on the baseline norm. Despite this, such behaviour is non-monotonous: when $\gamma =1$, cooperation levels under the emotion-based norm plummet. Each data point averages the results of 300 runs with the same parameter configuration. Other parameters: $b=5, c=1, z=50, \mu =1/z, \beta =1$.

The cooperation index $\eta$ promoted by EBIS and IS is shown in Fig. 1, as a function of $\gamma$, in an environment where errors are present (each error occurs on average once every 500 interactions). The main finding, confirming our expectations, was that cooperation was higher, when compared to the baseline norm (IS), the higher the probability that emotion expressions were considered. As a sanity check, notice the cooperation level promoted by IS is (a) consistent with prior work on this norm, in environments where errors are present, and unfazed by varying $\gamma$ values; and (b) how for $\gamma =0$, the performance of both norms is essentially identical. Interestingly, cooperation was not higher for IS in the extreme case of very high values of $\gamma$ (between $\approx 0.95$, and 1), where we face a cusp-like behaviour for this parameter—we analyze this further below.

**Fig. 2:**
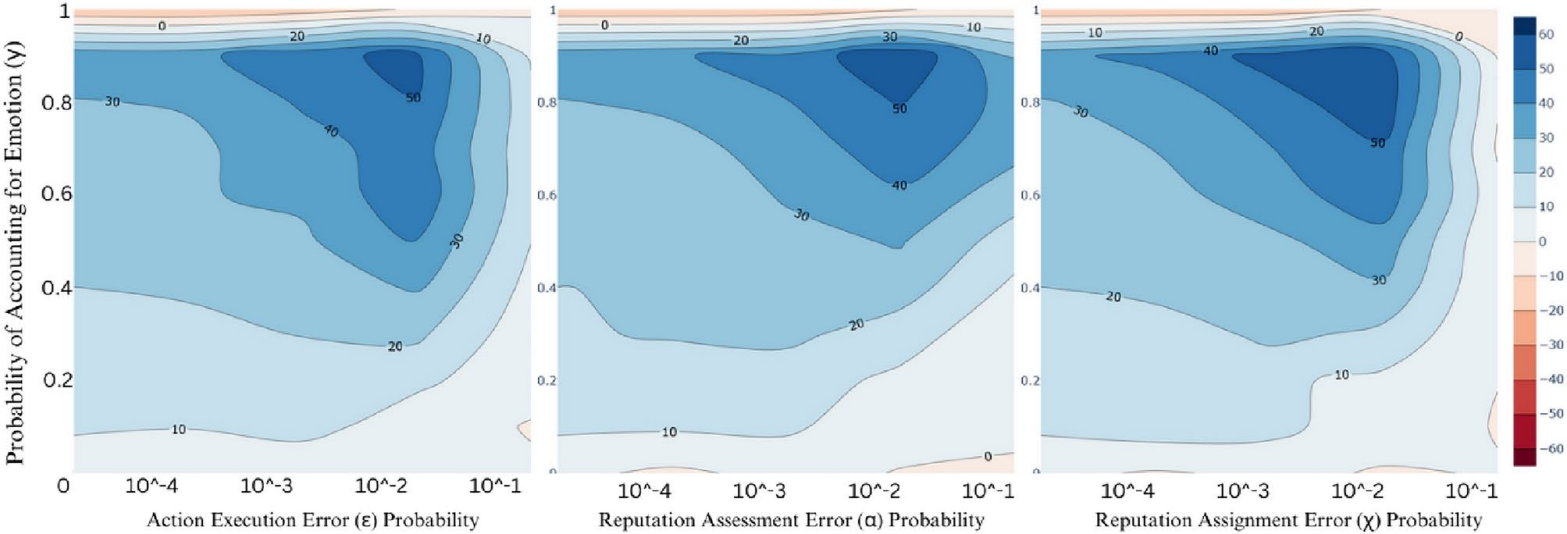
Contourplots of cooperation by error magnitude, for each of the studied errors $\varepsilon , \alpha , \chi$, respectively. Encoded by colour, according to the colour bar on the right-hand side, we plot the cooperation index difference between emotion-based image scoring and basic image scoring, by $\gamma$ value on the y-axis and by error magnitude on the x-axis, from among the following values: $\varepsilon , \chi , \alpha \in \{\frac{10^{-3}}{z}, \frac{10^{-2}}{z}, \frac{10^{-1}}{z}, \frac{1}{z}, \frac{10}{z}\}$, where *z* is the population size. For high $\gamma$ values, we can see that the greater the magnitude of each error, the greater the impact of using an emotion-based social norm, with two notable exceptions: when errors become too frequent ($\varepsilon =\chi = \alpha = \frac{10}{z} = 0.2$—one error occurring on average in every 5 interactions), and when $\gamma =1$—once again showing a phase transition occurring in the interval of $0.9 \le \gamma \le 1$. Each data point averages the results of 100 simulations. Other parameters are set to the same values as the previous figure.

Figure 2 consists of three contour plots, depicting the effects of each isolated error on cooperation levels, by varying the magnitude of each error. The diverging-scale colour-coded feature measures the cooperation index $\eta$ difference between EBIS and IS. Towards blue, we see positive differences and towards red negative ones, while the closer the colour is to white the smaller the difference in the cooperation levels. We can start by observing how, for $\gamma =0$, there is no significant difference between the cooperation promoted by both social norms. This is an expected result, since the smaller the probability emotion expressions are considered for the social norm, the more identical the two norms become. Another noticeable effect is again the presence of the phase-transition for values of $\gamma$ between 0.9 and 1, as was present in Fig. 1. Finally, while the power of a simple emotion-based social norm (EBIS) is already evident from the previous figure, the results from Fig. 2 show how EBIS is especially effective when in the presence of errors, judging by the darker blue shade around an error probability of $\frac{1}{z} = 0.02$ (which translates to one execution, assessment or assignment error, respectively, occurring in average every 50 interactions). For medium to high $\gamma$ values, EBIS shows higher resilience to high reputation assessment errors than to the other two types, whose cooperation levels drop close to the same values promoted by IS when approaching the extremely noisy environment of $\chi =0.2$.

**Fig. 3:**
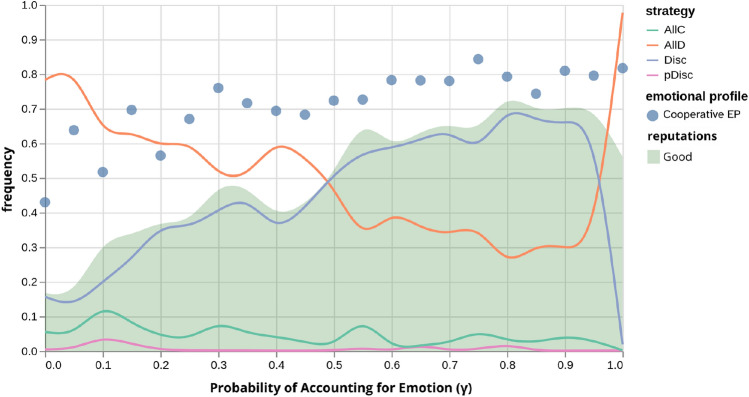
Average relative frequencies of emotional profiles, reputations and strategies by $\gamma$, for the emotion-based image scoring norm. Blue circles represent the average frequency of cooperative emotional profiles (complemented by the omitted frequency of competitive emotional profiles). Lines represent the frequency of the four possible strategies—always cooperate, always defect, discriminate and paradoxically discriminate. The green shaded area represents the average frequency of good reputations (complemented by the omitted frequency of bad reputations). One can observe how the dominance of the discriminate strategy, the cooperative emotional profile and the prevalence of good reputations for high $\gamma$ values steers the system towards the cooperation levels observed in Fig. 1. Error probabilities are set to values of $\varepsilon =\chi =\alpha =\frac{10^{-2}}{z}=0.002$, and all other parameters are set to the same values as the previous figure.

To understand what lies behind the so-called cusp behaviour, we plot on Fig. 3 the average frequency of strategies, reputations and emotional profiles by $\gamma$, under EBIS, resulting from the model’s evolutionary dynamics (see SI). The average frequency of the cooperative EP grows approximately linearly with $\gamma$. Symmetrically, since these frequencies complement each other, the average frequency of the competitive EP decreases. This indicates the existence of a direct relation between the dominance of the cooperative EP and the probability of considering emotion expression on moral evaluation. Meanwhile, the two most dominant strategies Disc—cooperate only with good individuals—and AllD—always defect—show a similar behaviour: Disc becomes more popular and AllD less frequent with greater $\gamma$ values. Overall, it appears that, concomitantly with cooperation levels, the evolutionary dynamics of the model select for a combination of cooperative strategies and cooperative EPs. Furthermore, the frequency of good reputations in the population closely follows the frequency of cooperative strategies. Once again, this ceases to be true for $\gamma =1$, where the cooperative EP no longer predicts the dominant strategy. In fact, we see a dominance of defective strategies for this extreme scenario, whereas emotional profiles (and reputations) become essentially meaningless. Interestingly, the system ceases to exert selective pressure on emotional profiles when emotion expressions are always contemplated by the social norm. Additionally, the low level of $\eta$ for $\gamma =1$ is linked to the drop in relative frequency of cooperative strategies (and consequent dominance of AllD). So why is cooperation unable to evolve in such a scenario? One explanation is that when $\gamma =1$, the evolutionarily stable and mathematically rational trait is that of not cooperating when displaying cooperative emotions, since the social norm fails to condemn this behaviour with a bad reputation, should the individual already be regarded as good (see Table 1). In other words, without having to pay cooperation-associated costs, emotionally cooperative defectors reap the benefits of cooperators since their reputation signals them as potential (indirect) reciprocators, rendering reputation-based cooperation ineffective. Once the population converges to an AllD-dominated state, reputations become meaningless (since this strategy does not discern between reputations), making in turn EPs useless in the face of an emotion-based social norm.

## Discussion

This paper highlights a gap in the study of reputation-based cooperation through models of evolutionary game theory: the modelling of emotion expressions as shapers of moral evaluations. We build on top of the classical model of Ohtsuki and Iwasa^19^ and a recent empirical study^20^ linking reputation assessment rules to emotional expressions in a prisoner’s dilemma. Based on these, we propose a computational and evolutionary approach to modelling moral emotions within the framework of indirect reciprocity. We do so by allowing actions between agents in the prisoner’s dilemma to be morally judged not only by their action and the relevant agent’s reputation—as is often modelled in the study of reputation-based cooperation^17–19^ but also, with probability $\gamma$, by the agents’ expressed emotions as reactions to the outcome of their joint actions in the prisoner’s dilemma. Our findings show that incorporating emotional expressions into moral assessments significantly improves cooperation levels compared to baseline norms that disregard emotions. Furthermore, we studied the effects of emotion-based moral judgements on noisy environments with three widely studied errors in the context of IR models^46^ action execution, reputation assessment and reputation assignment errors. While Image Scoring is known to be a simple norm capable of achieving cooperative states^10^ albeit only in the absence of errors^17^, our results suggest that in such noisy scenarios, modifying this norm to distinguish between the emotional expressions of cooperators and defectors allows for highly cooperative states, highlighting the impact emotional expressions have as intention-confirming and error-correcting mechanisms. We name this norm Emotion-Based Image Scoring.

Our findings also indicate that the system evolves towards a state of synchrony between cooperative strategies and emotional profiles. As a result, a known cooperator is not condemned to a bad reputation upon committing a single mistake - executing an isolated action that goes against their beliefs or intentions - as their expressed emotions signal to the population the agent’s real intention in cooperating. Such a population is not invaded by individuals attempting to exploit this social norm, such as defectors expressing cooperative emotions, since there are enough interactions that disregard emotion expressions, situations in which such a free-rider is punished with a bad reputation regardless of their EP. The dominant synchronization of cooperative EPs and strategies allows for cooperation levels highly robust to errors: the more emotion expressions are considered, the higher the cooperation level in the face of frequent errors, with the notable exception of $\gamma =1$. Moreover, this synchronization also addresses how EBIS deals with IS’s so-called *scoring dilemma*, whereby discriminant individuals’ reputations are harmed by punishing bad individuals, making discriminating strategies evolutionarily unstable: under EBIS, good individuals who punish bad players keep their social status as long as they express cooperation-aligned emotions. This trait synchronization is dominant for higher probabilities of emotion accountability ($\gamma$ values), as can be observed in Fig. 3.

What accounts for the cusp-like behaviour in cooperation levels for a perfect level of $\gamma$, i.e., always accounting for emotional expressions when morally assessing an interaction? This result reflects an unrealistic setting where the wide majority of individuals consistently mask defective intentions with cooperative emotion expressions. In such a world, it is perfectly acceptable to assume that individuals should quickly learn such behaviour as the optimal and rational strategy, even if incapable of fostering any cooperation between them: why would you not benefit from exploiting others if *pretending* to regret it was enough to make you a good person in the eyes of others? Reflecting insights from the study of signalling, namely those of cheap talk^50^, or apology and forgiveness^51^, if being devoid of any costs whilst having (indirect) impacts on payoffs, what incentive is there to be truthful? Signalling theory poses that, on the one hand, all signals, except for verbal communication, evolved to be reliable^52^; and on the other hand, that for a signal to be evolutionarily preserved in the population, the interaction should be beneficial for both sender and receiver^53^. Furthermore, some would argue that what tells emotion expressions and cheap talk apart is that the former is more involuntary and harder to fake than the latter, thus being considered by some as “honest signals”^48,49,54,55^, often regarded as considerably harder to fake^56^. Moreover, humans are remarkably poor at detecting cheaters through facial expression^57^, especially among strangers^58,59^, suggesting that either such a mechanism provides no fitness benefits and thus has never been subject to selective pressure^52,60^ or that the relevant ecological context for cheater detection through facial expression lies in social group members and not among strangers^32^, where IR is typically applied. Thus, this scenario of widespread dishonesty seems highly improbable: whereas humans are believed to possess the unique capacity to regulate or even portray emotions they do not feel to attain goals^22,61,62^, our findings suggest that if our social brains evolved to produce populations dominated by either total naiveté or ubiquitous “deceitism”, cooperative societies would never flourish. Our findings support the idea that while emotional expressions are useful and effective in helping assess the moral value of actions, widespread cooperation requires the ability to occasionally disregard them, or else a gene for emotion-faking might have become dominant. This might become more obvious by lifting a simplifying assumption our model makes, that of a universal and fixed $\gamma$ value for the whole population: by allowing individuals to heterogeneously evolve such an attribute through natural selection, the model might provide greater insights into to what extent individuals incorporate emotion expressions into moral evaluation. Inspired by previous work on diversity and cooperation, we propose that cognitive diversity in the individual’s ability to accurately express and recognise emotion expressions might be nature’s solution to the bleak scenario we expose for $\gamma =1$^63^.

Our work provides evolutionary support for Darwin’s hypothesis that emotional expressions evolved to facilitate communication, paramount to the construction of cooperative relationships. Moreover, we provide an open research avenue for future work on the roles of emotional expression in IR. Our model can be extended to produce exhaustive research on all possible social norms of similar structure, eventually highlighting a new panorama of *leading-eight* donor-focused emotion-based social norms capable of fostering cooperation. Emotion expressions can also be leveraged to facilitate information-dissemination problems common to IR^27,39,64,65^, alongside other dimensions of social intelligence that overcome such problems, such as empathy^66,67^ and pleasing^68^ on moral assessment. Finally, our research has practical applications for designing complex systems like autonomous agents, social robots, and other artificially intelligent systems. It can guide these systems in shaping human behaviour towards cooperation and pro-sociality^69–71^ and in fostering trust^72^. This includes not only using reputation systems and appropriate behaviours but also leveraging nonverbal cues, such as emotional expressions, to confirm intentions and correct mistakes.

## Limitations of the study

One limitation of our model exists in its connection to IR itself: our donor-focused approach to social norms does not make use of one of the commonly studied features of reputation-based cooperation, since, in our model, the reputation of a recipient determines how the potential donor should act but is overlooked on the judgement of said donor’s action. Thus, the commonly studied second-order complexity^17–19^ of how to evaluate actions towards good or bad individuals is lost. Regardless, as this model is easily extendable to the study of the classical recipient-focused second-order social norms, we underscore that the main findings from this research—that emotion expressions can play a crucial role in helping sustain reputation-based cooperation—are extendable to a number of such norms. Our preliminary analysis of emotion-based variations of widely studied norms, as described in the Supplementary Information, suggests that some emotion-discriminating changes, akin to the ones applied in this work, greatly benefit cooperation levels. How to change these norms to better support cooperation, however, is not trivial. For example, the second-order flagship of IR, Stern Judging (SJ), shows such an impressive resilience to errors^18,38^ that any alteration—such as discriminating between emotion expressions—fails to produce any positive effect. Furthermore, other more complex forms of moral evaluation have also been studied, such as merging IR with group reciprocity^73^, opening the door for research on how emotions expressed within groups might affect group-based cooperation. We propose such extensive analysis of more complex emotion-based social norms for future work, accompanied by appropriate user studies focusing on this classical framework.

## Supplementary Information

Supplementary Material 1.

## Data Availability

All generated data and the code supporting the model are available in the following public repository: https://github.com/cfonsecahenrique/SNARE.

## References

[1] Pennisi, E. How did cooperative behavior evolve?. *Science* **309**, 93–93. 10.1126/science.309.5731.93 (2005). 15994539 10.1126/science.309.5731.93

[2] Rand, D. G. & Nowak, M. A. Human cooperation. *Trends Cogn. Sci.* **17**, 413–425. 10.1016/j.tics.2013.06.003 (2013). 23856025 10.1016/j.tics.2013.06.003

[3] Traulsen, A. & Glynatsi, N. E. The future of theoretical evolutionary game theory. *Philos. Trans. R. Soc. B: Biol. Sci.*[SPACE]10.1098/rstb.2021.0508 (2023). 10.1098/rstb.2021.0508 PMC10024985 36934760

[4] Maynard Smith, J. *Evolution and the Theory of Games* (Cambridge University Press, Cambridge, 1982).

[5] Alexander, R. D. *The Biology of Moral* Systems1st edn. (Routledge, London, 2017).

[6] Mailath, G. J. Introduction: Symposium on evolutionary game theory. *J. Econ. Theory* **57**, 259–277. 10.1016/0022-0531(92)90036-H (1992).

[7] Zaggl, M. A. Eleven mechanisms for the evolution of cooperation. *J. Inst. Econ.* **10**, 197–230. 10.1017/S1744137413000374 (2014).

[8] Trivers, R. L. The evolution of reciprocal altruism. *Q. Rev. Biol.* **46**, 35–57. 10.1086/406755 (1971).

[9] Axelrod, R. & Hamilton, W. D. The evolution of cooperation. *Science* **211**, 1390–1396. 10.1126/science.7466396 (1981). 7466396 10.1126/science.7466396

[10] Nowak, M. A. & Sigmund, K. Evolution of indirect reciprocity by image scoring. *Nature* **393**, 573–577. 10.1038/31225 (1998). 9634232 10.1038/31225

[11] Nowak, M. A. & Sigmund, K. Evolution of indirect reciprocity. *Nature* **437**, 1291–1298. 10.1038/nature04131 (2005). 16251955 10.1038/nature04131

[12] Roberts, G. Evolution of direct and indirect reciprocity. *Proc. R. Soc. B: Biol. Sci.* **275**, 173–179. 10.1098/rspb.2007.1134 (2008). 10.1098/rspb.2007.1134 PMC2596181 17971326

[13] Milinski, M., Semmann, D. & Krambeck, H.-J. Reputation helps solve the ‘tragedy of the commons’. *Nature* **415**, 424–426. 10.1038/415424a (2002). 11807552 10.1038/415424a

[14] Boyd, R. & Richerson, P. J. The evolution of indirect reciprocity. *Soc. Netw.* **11**, 213–236. 10.1016/0378-8733(89)90003-8 (1989).

[15] Kandori, M. Social norms and community enforcement. *Rev. Econ. Stud.* **59**, 63. 10.2307/2297925 (1992).

[16] Leimar, O. & Hammerstein, P. Evolution of cooperation through indirect reciprocity. *Proc. R. Soc. Lond. Ser. B: Biol. Sci.* **268**, 745–753. 10.1098/rspb.2000.1573 (2001). 10.1098/rspb.2000.1573 PMC1088665 11321064

[17] Panchanathan, K. & Boyd, R. A tale of two defectors: the importance of standing for evolution of indirect reciprocity. *J. Theor. Biol.* **224**, 115–126. 10.1016/S0022-5193(03)00154-1 (2003). 12900209 10.1016/s0022-5193(03)00154-1

[18] Pacheco, J. M., Santos, F. C. & Chalub, F. A. C. C. Stern-judging: A simple, successful norm which promotes cooperation under indirect reciprocity. *PLoS Comput. Biol.* **2**, e178. 10.1371/journal.pcbi.0020178 (2006). 17196034 10.1371/journal.pcbi.0020178 PMC1761656

[19] Ohtsuki, H. & Iwasa, Y. How should we define goodness?—reputation dynamics in indirect reciprocity. *J. Theor. Biol.* **231**, 107–120. 10.1016/j.jtbi.2004.06.005 (2004). 15363933 10.1016/j.jtbi.2004.06.005

[20] de Melo, C. M., Terada, K. & Santos, F. C. Emotion expressions shape human social norms and reputations. *iScience* **24**, 102141. 10.1016/j.isci.2021.102141 (2021). 33665560 10.1016/j.isci.2021.102141 PMC7898177

[21] de Melo, C. M., Gratch, J., Marsella, S. & Pelachaud, C. Social functions of machine emotional expressions. *Proc. IEEE* **111**, 1382–1397. 10.1109/JPROC.2023.3261137 (2023).

[22] van Kleef, G. A. & Côté, S. The social effects of emotions. *Annu. Rev. Psychol.* **73**, 629–658. 10.1146/annurev-psych-020821-010855 (2022). 34280326 10.1146/annurev-psych-020821-010855

[23] Szolnoki, A., Xie, N.-G., Wang, C. & Perc, M. Imitating emotions instead of strategies in spatial games elevates social welfare. *EPL Europhys. Lett.* **96**, 38002. 10.1209/0295-5075/96/38002 (2011).

[24] Szolnoki, A., Xie, N.-G., Ye, Y. & Perc, M. Evolution of emotions on networks leads to the evolution of cooperation in social dilemmas. *Phys. Rev. E* **87**, 042805. 10.1103/PhysRevE.87.042805 (2013). 10.1103/PhysRevE.87.042805 23679471

[25] Chen, W. et al. Effects of emotion on the evolution of cooperation in a spatial prisoner’s dilemma game. *Appl. Math. Comput.* **411**, 126497. 10.1016/j.amc.2021.126497 (2021).

[26] Bai, X., Ye, Y., Chen, T. & Xie, N. The evolutionary game of emotions considering the influence of reputation. *Appl. Math. Comput.* **474**, 128709. 10.1016/j.amc.2024.128709 (2024).

[27] Okada, I. A review of theoretical studies on indirect reciprocity. *Games* **11**, 27. 10.3390/g11030027 (2020).

[28] Frank, R. H. *Introducing Moral Emotions into Models of Rational Choice* 422–440 (Cambridge University Press, Cambridge, 2004).

[29] Boone, R. T. & Buck, R. Emotional expressivity and trustworthiness: The role of nonverbal behavior in the evolution of cooperation. *J. Nonverbal Behav.* **27**, 163–182. 10.1023/A:1025341931128 (2003).

[30] Darwin, C. *The expression of the emotions in man and animals.* (John Murray, 1872).

[31] van Kleef, G. A. *The Interpersonal Dynamics of Emotion* (Cambridge University Press, Cambridge, 2016).

[32] Schmidt, K. L. & Cohn, J. F. Human facial expressions as adaptations: Evolutionary questions in facial expression research. *Am. J. Phys. Anthropol.* **116**, 3–24. 10.1002/ajpa.20001 (2001). 10.1002/ajpa.2001 PMC2238342 11786989

[33] Fridlund, A. J. Human facial expression: An evolutionary view. *Choice Rev. Online* **32**, 3890. 10.5860/CHOICE.32-3890 (1995).

[34] Fischer, A.H. & Manstead, A. S.R. *Social functions of emotion*, 456–468 (The Guilford Press, 2008).

[35] de Melo, C. M., Carnevale, P. J., Read, S. J. & Gratch, J. Reading people’s minds from emotion expressions in interdependent decision making. *J. Pers. Soc. Psychol.* **106**, 73–88. 10.1037/a0034251 (2014). 24079297 10.1037/a0034251

[36] de Melo, C. M. & Terada, K. The interplay of emotion expressions and strategy in promoting cooperation in the iterated prisoner’s dilemma. *Sci. Rep.* **10**, 14959. 10.1038/s41598-020-71919-6 (2020). 32917943 10.1038/s41598-020-71919-6 PMC7486426

[37] Fridlund, A. J. *The New Ethology of Human Facial Expressions* 103–130 (Cambridge University Press, Cambridge, 1997).

[38] Santos, F. P., Pacheco, J. M. & Santos, F. C. Evolution of cooperation under indirect reciprocity and arbitrary exploration rates. *Sci. Rep.* **6**, 37517. 10.1038/srep37517 (2016). 27892509 10.1038/srep37517 PMC5124964

[39] Hilbe, C., Schmid, L., Tkadlec, J., Chatterjee, K. & Nowak, M. A. Indirect reciprocity with private, noisy, and incomplete information. *Proc. Natl. Acad. Sci.* **115**, 12241–12246. 10.1073/pnas.1810565115 (2018). 30429320 10.1073/pnas.1810565115 PMC6275544

[40] Brandt, H. & Sigmund, K. The good, the bad and the discriminator—errors in direct and indirect reciprocity. *J. Theor. Biol.* **239**, 183–194. 10.1016/j.jtbi.2005.08.045 (2006). 16257417 10.1016/j.jtbi.2005.08.045

[41] Ohtsuki, H. & Iwasa, Y. The leading eight: Social norms that can maintain cooperation by indirect reciprocity. *J. Theor. Biol.* **239**, 435–444. 10.1016/j.jtbi.2005.08.008 (2006). 16174521 10.1016/j.jtbi.2005.08.008

[42] Ohtsuki, H., Iwasa, Y. & Nowak, M. A. Reputation effects in public and private interactions. *PLoS Comput. Biol.* **11**, e1004527. 10.1371/journal.pcbi.1004527 (2015). 26606239 10.1371/journal.pcbi.1004527 PMC4659694

[43] Dunbar, R. *Grooming, Gossip and the Evolution of Language* (Harvard University Press, 1996).

[44] Sommerfeld, R. D., Krambeck, H.-J., Semmann, D. & Milinski, M. Gossip as an alternative for direct observation in games of indirect reciprocity. *Proc. Natl. Acad. Sci.* **104**, 17435–17440. 10.1073/pnas.0704598104 (2007). 17947384 10.1073/pnas.0704598104 PMC2077274

[45] Fishman, M. A. Indirect reciprocity among imperfect individuals. *J. Theor. Biol.* **225**, 285–292. 10.1016/S0022-5193(03)00246-7 (2003). 14604582 10.1016/s0022-5193(03)00246-7

[46] Santos, F. P., Santos, F. C. & Pacheco, J. M. Social norm complexity and past reputations in the evolution of cooperation. *Nature* **555**, 242–245. 10.1038/nature25763 (2018). 29516999 10.1038/nature25763

[47] Santos, F. P., Pacheco, J. M. & Santos, F. C. The complexity of human cooperation under indirect reciprocity. *Philos. Trans. R. Soc. B: Biol. Sci.*[SPACE]10.1098/rstb.2020.0291 (2021). 10.1098/rstb.2020.0291 PMC8487734 34601904

[48] Centorrino, S., Djemai, E., Hopfensitz, A., Milinski, M. & Seabright, P. Honest signaling in trust interactions: Smiles rated as genuine induce trust and signal higher earning opportunities. *Evol. Hum. Behav.* **36**, 8–16. 10.1016/j.evolhumbehav.2014.08.001 (2015).

[49] Reed, L. I., Zeglen, K. N. & Schmidt, K. L. Facial expressions as honest signals of cooperative intent in a one-shot anonymous prisoner’s dilemma game. *Evol. Hum. Behav.* **33**, 200–209. 10.1016/j.evolhumbehav.2011.09.003 (2012).

[50] Farrell, J. & Rabin, M. Cheap talk. *J. Econ. Perspect.* **10**, 103–118. 10.1257/jep.10.3.103 (1996).

[51] Martinez-Vaquero, L. A., Han, T. A., Pereira, L. M. & Lenaerts, T. Apology and forgiveness evolve to resolve failures in cooperative agreements. *Sci. Rep.* **5**, 10639. 10.1038/srep10639 (2015). 26057819 10.1038/srep10639 PMC4460819

[52] Zahavi, A. The fallacy of conventional signalling. *Philos. Trans. R. Soc. Lond. Ser. B: Biol. Sciences* **340**, 227–230. 10.1098/rstb.1993.0061 (1993). 8101657 10.1098/rstb.1993.0061

[53] Pentland, A. *Honest Signals* (The MIT Press, 2008).

[54] Reed, L. I., Matari, Y., Wu, M. & Janaswamy, R. Emotional tears: An honest signal of trustworthiness increasing prosocial behavior?. *Evol. Psychol.*[SPACE]10.1177/1474704919872421 (2019). 31455105 10.1177/1474704919872421 PMC10299780

[55] Wacewicz, S. & Zywiczynski, P. Human honest signalling and nonverbal communication. *Psychol. Lang. Commun.* **16**, 113–130. 10.2478/v10057-012-0009-5 (2012).

[56] Keltner, D. & Bonanno, G. A. A study of laughter and dissociation: Distinct correlates of laughter and smiling during bereavement. *J. Pers. Soc. Psychol.* **73**, 687–702. 10.1037/0022-3514.73.4.687 (1997). 9325589 10.1037//0022-3514.73.4.687

[57] Frank, M. G. & Ekman, P. The ability to detect deceit generalizes across different types of high-stake lies. *J. Pers. Soc. Psychol.* **72**, 1429–1439. 10.1037/0022-3514.72.6.1429 (1997). 9177024 10.1037//0022-3514.72.6.1429

[58] DePaulo, B. M. Nonverbal behavior and self-presentation. *Psychol. Bull.* **111**, 203–243. 10.1037/0033-2909.111.2.203 (1992). 1557474 10.1037/0033-2909.111.2.203

[59] Gosselin, P., Kirouac, G. & Doré, F. Y. Components and recognition of facial expression in the communication of emotion by actors. *J. Pers. Soc. Psychol.* **68**, 83–96. 10.1037/0022-3514.68.1.83 (1995). 7861316 10.1037//0022-3514.68.1.83

[60] Bradbury, J. W. & Vehrencamp, S. L. Economic models of animal communication. *Anim. Behav.* **59**, 259–268. 10.1006/anbe.1999.1330 (2000). 10675247 10.1006/anbe.1999.1330

[61] van Kleef, G. A. How emotions regulate social life. *Curr. Dir. Psychol. Sci.* **18**, 184–188. 10.1111/j.1467-8721.2009.01633.x (2009).

[62] Fridlund, A. J. Evolution and facial action in reflex, social motive, and paralanguage. *Biol. Psychol.* **32**, 3–100. 10.1016/0301-0511(91)90003-Y (1991). 1742403 10.1016/0301-0511(91)90003-y

[63] Santos, F. C., Pinheiro, F. L., Lenaerts, T. & Pacheco, J. M. The role of diversity in the evolution of cooperation. *J. Theor. Biol.* **299**, 88–96. 10.1016/j.jtbi.2011.09.003 (2012). 21930134 10.1016/j.jtbi.2011.09.003

[64] Uchida, S. Effect of private information on indirect reciprocity. *Phys. Rev. E* **82**, 036111. 10.1103/PhysRevE.82.036111 (2010). 10.1103/PhysRevE.82.036111 21230143

[65] Uchida, S. & Sasaki, T. Effect of assessment error and private information on stern-judging in indirect reciprocity. *Chaos, Solitons Fractals* **56**, 175–180. 10.1016/j.chaos.2013.08.006 (2013).

[66] Radzvilavicius, A. L., Stewart, A. J. & Plotkin, J. B. Evolution of empathetic moral evaluation. *eLife* **8**, 1–18. 10.7554/eLife.44269 (2019). 10.7554/eLife.44269 PMC6488294 30964002

[67] Masuda, N. & Santos, F. C. A mathematical look at empathy. *eLife*[SPACE]10.7554/eLife.47036 (2019). 31032800 10.7554/eLife.47036 PMC6488291

[68] Krellner, M. & Han, T. A. Pleasing enhances indirect reciprocity-based cooperation under private assessment. *Artif. Life* **27**, 246–276. 10.1162/artl_a_00344 (2022).

[69] Paiva, A., Santos, F. & Santos, F. Engineering pro-sociality with autonomous agents. *Proc. AAAI Conf. Artif. Intell.* **32**, 7994–7999. 10.1609/aaai.v32i1.12215 (2018).

[70] de Melo, C. M., Gratch, J., Marsella, S. & Pelachaud, C. Social functions of machine emotional expressions. *Proc. IEEE* **111**, 1382–1397. 10.1109/JPROC.2023.3261137 (2023).

[71] Crandall, J. W. et al. Cooperating with machines. *Nat. Commun.* **9**, 233. 10.1038/s41467-017-02597-8 (2018). 29339817 10.1038/s41467-017-02597-8 PMC5770455

[72] de Melo, C. M., Marsella, S. & Gratch, J. Human cooperation when acting through autonomous machines. *Proc. Natl. Acad. Sci.* **116**, 3482–3487. 10.1073/pnas.1817656116 (2019). 30808742 10.1073/pnas.1817656116 PMC6397531

[73] Nax, H. H., Perc, M., Szolnoki, A. & Helbing, D. Stability of cooperation under image scoring in group interactions. *Sci. Rep.* **5**, 12145. 10.1038/srep12145 (2015). 26177466 10.1038/srep12145 PMC4502532

