# Supplementary material for: Evolution of indirect reciprocity under emotion expression
Source: Sci Rep. 2025 Mar 17;15:9151. doi: 10.1038/s41598-025-89588-8 (PMC11914290; doi:10.1038/s41598-025-89588-8)
Supplement: Supplementary file 1 — Supplementary Material 1. [file 41598_2025_89588_MOESM1_ESM.pdf]

Supplementary Materials for

Evolution of Indirect Reciprocity under Emotion Expression

Henrique Correia da Fonseca *et al.*

*Corresponding author. Email: henrique.c.fonseca@tecnico.ulisboa.pt

This PDF file includes:

Supporting text
Figures S1 to S6
SI References

Supplementary Text
Methods

Here we summarize the computational model and mathematical methods.

Actions conditional on reputations. The action of agent players in the prisoner's dilemma depends on the current reputation of the opposite player, as usually implemented in Indirect Reciprocity (IR) literature (Brandt and Sigmund, 2006). Here, we consider reputation assessment errors with probability χ, whereby an individual retrieving another's public reputation fails to assess the correct image of this individual. Considering binary reputations (1='good'=G or 0='bad'=B), strategies in this model are 2-bit strings that prescribe an action (1='cooperate'=C or 0='defect'=D) on the basis of the other player's reputations. Thus, strategies can be represented as $P = (p_0, p_1)$ with $p_i \in \{0, 1\}$ denoting the action of a player towards agents with each possible reputation (0 or 1). This allows for 2^2=4 possible strategies, commonly known as *always defect* or *AllD* = (0,0); *paradoxical discriminator* or *pDisc* = (0,1); *discriminator* or *Disc* = (1,0); and *always cooperate* or *AllC* = (1,1). We consider execution errors (with probability ε) that represent the inability of individuals to act in the way their strategy dictates (Fishman, 2003).

Social norms and reputation dynamics. In our model, the new reputation of an individual is dictated, with probability γ, by an emotion-based social norm, or, with the complementary probability 1-γ, its classical emotion-agnostic version. The former can be written as $r' = (r_{gcn}, r_{gcm}, r_{gdn}, r_{gdm}, r_{bcn}, r_{bcm}, r_{bdn}, r_{bdm})$, whereas the latter are depicted as $r' = (r_{gc}, r_{gd}, r_{bc}, r_{bd})$. We consider reputation assignment errors with probability α, occurring when the observer fails to assign the correct reputation. Once a reputation is assigned, it is instantaneously and perfectly disseminated throughout the population – through mechanisms such as gossip (Sommerfeld et al, 2007) - so that information is fully consensual: all individuals share the same opinion regarding all other agents. These reputation dynamics are depicted in Fig. S1.

Evolutionary dynamics. Based on the seminal model of Ohtsuki and Iwasa (Ohtsuki and Iwasa, 2004), at the beginning of each simulation, each of z individuals adopts a random strategy and a random emotional profile (from 1 = *cooperative* or 0 = *competitive*) and is assigned a random reputation, all chosen using a uniform probability distribution. The tuple of strategy and emotional profile is henceforth referred to as the evolutionary trait. Simulations are run for a $g = 3000*z$ generations. For each generation, with probability $\mu = 1/z$, a random agent is selected to explore a new evolutionary trait, randomly mutating into a new strategy and/or emotional profile – a process well explored by Santos et al (Santos et al, 2016a). With the complementary probability 1-μ, two agents (X and Y) are randomly selected to play z prisoner's dilemma games (characterized as a two-way simultaneous donation game with b=5 and c=1) with randomly selected partners. After this Monte Carlo step, both agents compare their normalized fitness and learn the most successful evolutionary trait stochastically, with a probability given by $P(X \rightarrow Y) = (1 - e^{-\beta(f_Y - f_X)})^{-1}$, the so-called Fermi update or pairwise comparison rule (Traulsen et al, 2006), with β known as the intensity of selection (used throughout this manuscript as $\beta = 1$, see below analysis of other values) and f_X and f_Y standing for the normalized fitness value of agents X and Y, respectively. Allowing for the system to converge from its stochastic initialization, the first 10% of each simulation's generations are not recorded for the relevant metrics.

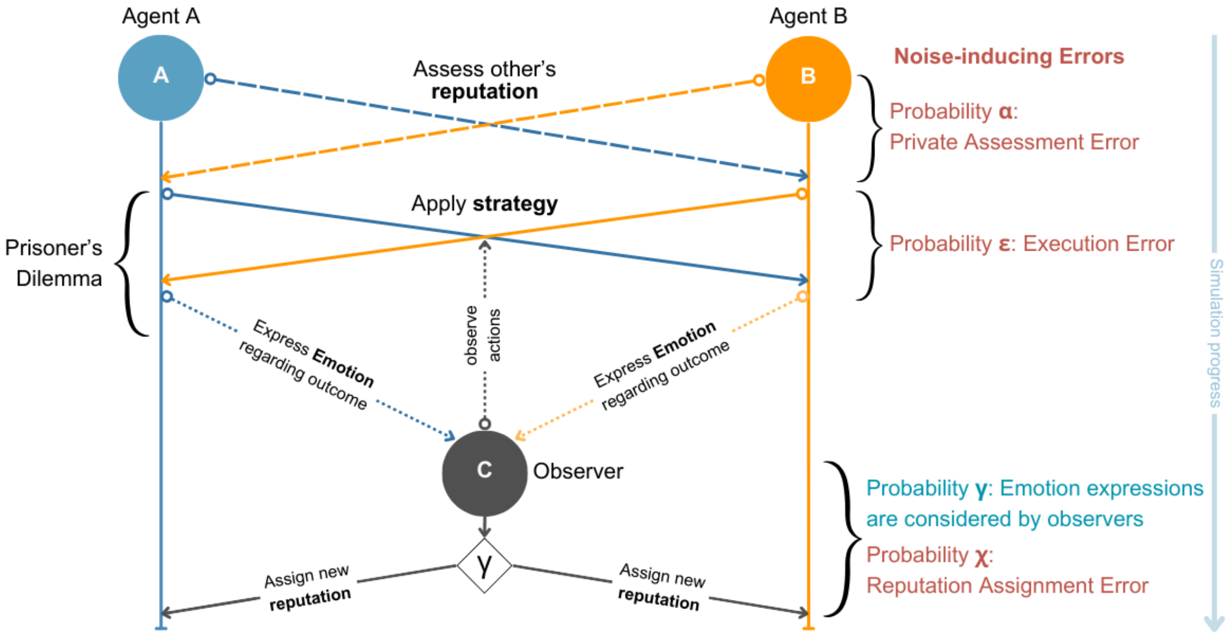

Fig. S1. Summary of reputation dynamics during an instance of the prisoner's dilemma.

Cooperation Index. The cooperation index η, also known as the average cooperation ratio, is computed as the fraction of cooperative acts that take place out of the total number of acts during the simulation time. Thus, η reflects both the dependence of strategy adoption on the relative frequency of strategies present in the population (frequency-dependent selection) and the evolution of reputations given the fixed (emotion-based or emotion-agnostic) social norms in the population.

Payoffs. The cooperation dilemma played by agents in our model is that of the prisoner's dilemma, with payoffs corresponding to a two-way simultaneous donation game with the cost of cooperation = 1 and benefit = 5, as usually modelled in the literature. Thus, the reward of mutual cooperation (R) is b-c=4, the sucker's payoff (S) translates into a personal cost of c=1, the temptation to defect (T) is the pure benefit b=5 and the punishment (P) of mutual defection 0. Although these were the values chosen for the experiments performed in the main manuscript, different benefit-to-cost ratios can be analysed, as displayed further below in this document.

Emotion Strict versus Lenient Image Scoring

Throughout the main manuscript, we study the workings of IR with a simple first-order social norm called Image Scoring (IS), which states that cooperative acts lead to good reputations and bad reputations otherwise (Nowak and Sigmund, 1998). To do this, we suggest two ways in which this social norm can be altered to discriminate between the emotions expressed by individuals upon playing the prisoner's dilemma. Reusing the formal notation for social norms described in the main manuscript, $r' = (r_{gcn}, r_{gcm}, r_{gdn}, r_{gdm}, r_{bcn}, r_{bcm}, r_{bdn}, r_{bdm})$, (i) emotion-lenient image scoring (ELIS) – (G, G, G, B, G, G, B, B) – prescribes that good donors who display regret after refusing to help their counterparts still deserve a good reputation; and (ii) emotion-strict image scoring (ESIS) – (G, G, G, B, G, B, B, B) – adding a clause to the previous norm stating that the only way bad individuals can increase their reputation is by confirming their cooperative intent with cooperation-aligned emotion expressions.

As shown in the main manuscript, ELIS is highly capable of supporting cooperation. ESIS displays a very similar behaviour, as can be seen in Figure S1, supporting more cooperation the higher the γ value, i.e. the higher the likelihood that emotion expressions are scrutinized by the social norm, with a steep decline when γ→1. Both clearly provide better avenues for cooperation to evolve than the baseline norm IS.

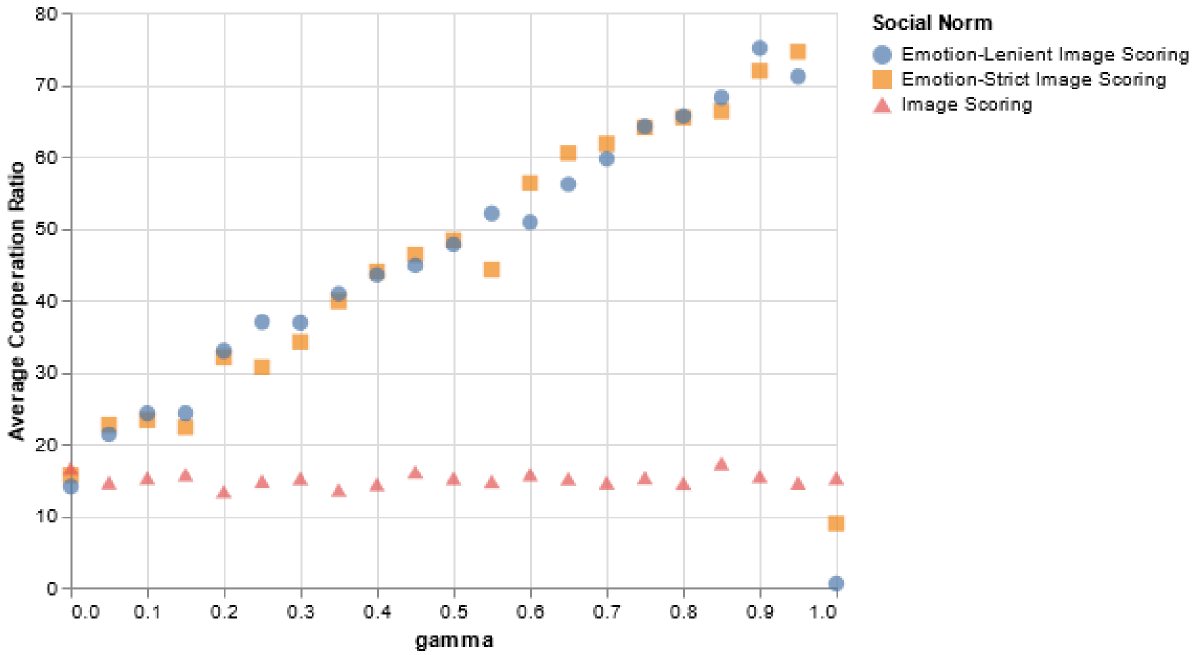

Fig. S2. Performance comparison of Emotion-Lenient Image Scoring (ELIS) and Emotion-Strict Image Scoring (ESIS) measured through the cooperation index η (or average cooperation ratio), for various γ probabilities of accounting for emotion. Orange squares represent values of η for ESIS, blue circles for ELIS and red triangles for basic IS. ELIS and EBIS show similar and significant increases in cooperation with higher γ values, with the exception of the cusp behaviour right before γ=1. IS remains constant for different γ values, as expected. Each datapoint corresponds to the average value of N=50 simulations. Other parameters: ε=α=χ=0.002, b/c=5, z=50, μ=1/z.

Regarding classic Second-Order Social Norms

Here we present results for emotion-based variations of the other classically studied second-order social norms, after modifying the model to use such recipient-focused norms, as done in the literature of second-order social norms (Pacheco et al, 2006; Santos et al 2016a; Santos et al 2016b). Thus, in this context, the g and b of the social norm vector $r' = (r_{gcn}, r_{gcm}, r_{gdn}, r_{gdm}, r_{bcn}, r_{bcm}, r_{bdn}, r_{bdm})$ correspond to the reputation of the recipient of the action, instead of that of the one acting.

There are different ways to make such a norm discriminate between emotion expressions. As we have seen before, norms can become stricter – imposing that the expressed emotions align themselves with the maxim of the norm – or more lenient – allowing for usually unsanctioned actions to be acceptable with the right emotion expression. There are even different levels in which a norm can become stricter or more lenient, for example: a highly strict variation of IS defined by the vector (G,B,B,G,G,B,B,G) would prescribe that both cooperation with good individuals and defection towards bad ones must be accompanied by cooperative emotion expressions, keeping a close eye on the intention signaled by the emotion expression (our preliminary analysis suggests this to be ineffective in supporting cooperation).

Thus, it is not trivial how to change these well-known norms in order to discriminate between emotion expressions while simultaneously retaining their own unique maxim and remaining as (or becoming more) effective in supporting cooperation. In the main manuscript, our approach to this problem was informed by a previous behavioral study (de Melo et al, 2021). Regarding the social norms followingly presented, whereas the chosen emotion-based variations help support the main idea of emotion expressions helping support cooperation, other variations can be computed that do not show such benefit. We leave such an extensive analysis of all possible variations of each norm, how to compute them, their effectiveness and robustness to each type of error, and what maxims these represent to a future work combining our computational approach with the appropriate behavioral study.

Shunning (SH)

In Figure S2 we show the benefits of considering emotion-expression under an emotion-based variation of Shunning (SH). SH states the only act worthy of a good reputation is that of helping a good individual, whereas all other actions yield bad reputations. Its studied emotion discriminating form – Emotion-Based Shunning (EBSH) (G,G,B,B,G,B,B,B) – prescribes the same idea, with the addendum that cooperating with a bad individual still warrants a good reputation, as long as the donor expresses cooperative emotions. This variation turns a highly prohibitive norm slightly more lenient, which benefits cooperation levels with higher γ values. Also note the lack of the cusp behavior seen for ESIS or ELIS.

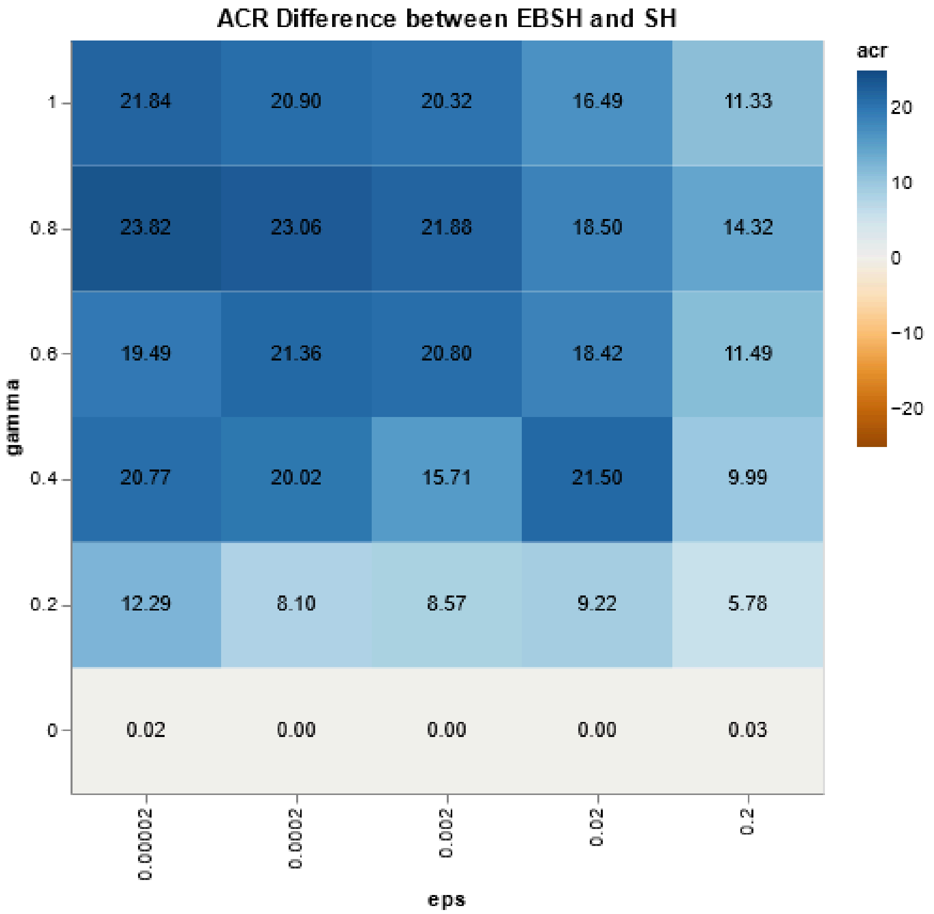

Fig. S3. Difference in cooperation index η between an Emotion-Based version of Shunning (EBSH) and Shunning (SH), for different magnitudes of execution errors and γ probabilities of accounting for emotion. Although the absolute values of η under EBSH remain somewhat low (between 20% and 35% of η), this Figure still indicates a significant increase in cooperation levels in comparison with SH. Each datapoint averages the value of η of N=50 independent simulations. α=χ=0.0002. Other parameters set to the same values of the previous Figure.

Simple Standing (SS)

Another widely studied social norm is that of Simple Standing (SS), prescribing that only the refusal of help towards a good individual warrants a bad reputation. Our studied Emotion-Based version of this norm (EBSS) (G,G,B,B,G,B,G,G), corrects this maxim stating that defection towards individuals with bad reputations must be confirmed with cooperative emotion expressions to be considered a good action. In Figure S3 we can see how beneficial these changes are to cooperation. Note once again the lack of a phase transition near $\gamma \rightarrow 1$ such as the one detailed in the main manuscript.

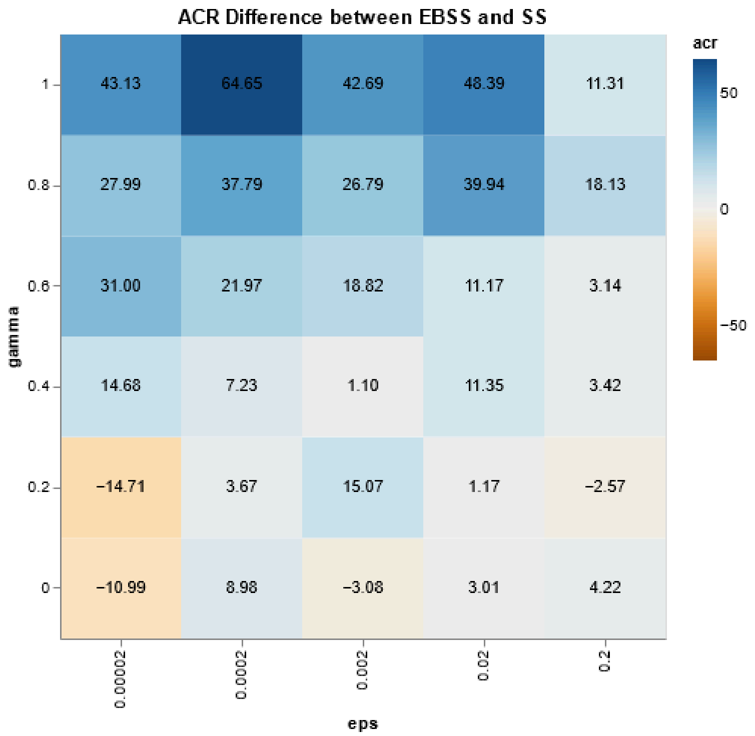

Fig. S4. Difference in cooperation index η between an Emotion-Based version of Simple Standing (EBSS) and Simple Standing (SS), for different magnitudes of execution errors and γ probabilities of accounting for emotion. The absolute values of η for EBSS reach significantly high values (such as >80% of η for ε=0.0002 and γ=1), suggesting that accounting for emotional profiles greatly benefits cooperation under this norm. Each datapoint averages the value of η of N=50 independent simulations. $\alpha=\chi=0.0002$. Other parameters set to the same values of previous Figures.

Stern Judging (SJ)

Stern Judging (SJ) is often considered the flagship of Indirect Reciprocity (Pacheco et al, 2006). It is highly resilient to errors (Santos et al, 2016b), creating a curious conundrum for the study of an emotion-based version of such norm: through our preliminary analysis, we could not find any emotion-based variation that would improve the already high levels of cooperation promoted by such norm, regardless of error magnitude. We posit, however, that such variations might play a role in supporting cooperation in scenarios in which SJ falters, such as those of private assessment and imperfect information (Uchida, 2010; Hilbe et al, 2018).

Robustness of main findings to other parameters

Benefit-to-cost ratio

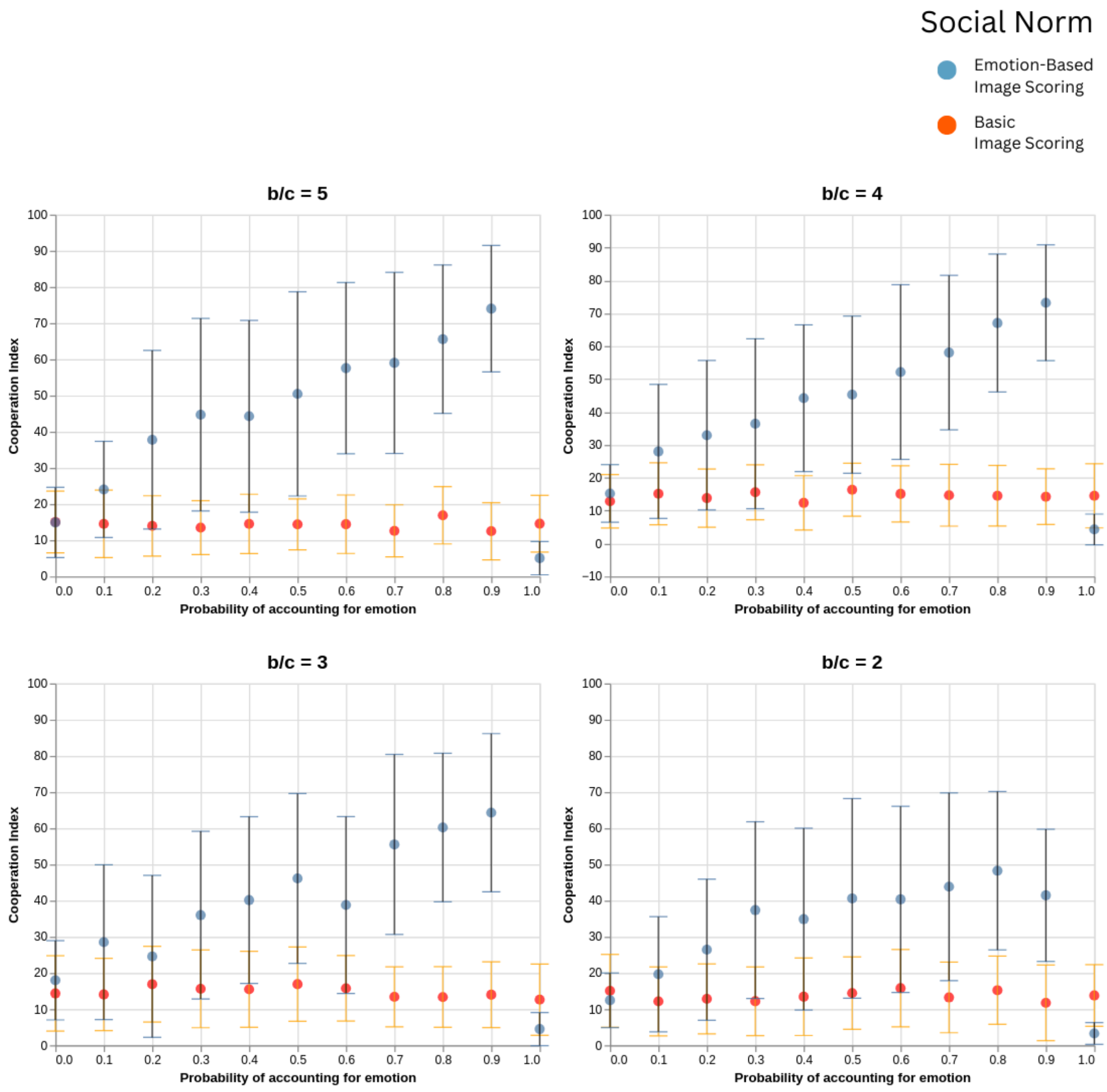

Fig. S5. Analysis of the robustness of cooperation promoted by EBIS to different benefit-to-cost ratios. One can observe how the main result – using emotion expressions generally leads to higher cooperation indexes – remains true for all studied benefit-to-cost ratios, a difference that becomes (expectedly) smaller with harder dilemmas (for lower benefit payoffs).

Intensity of Selection

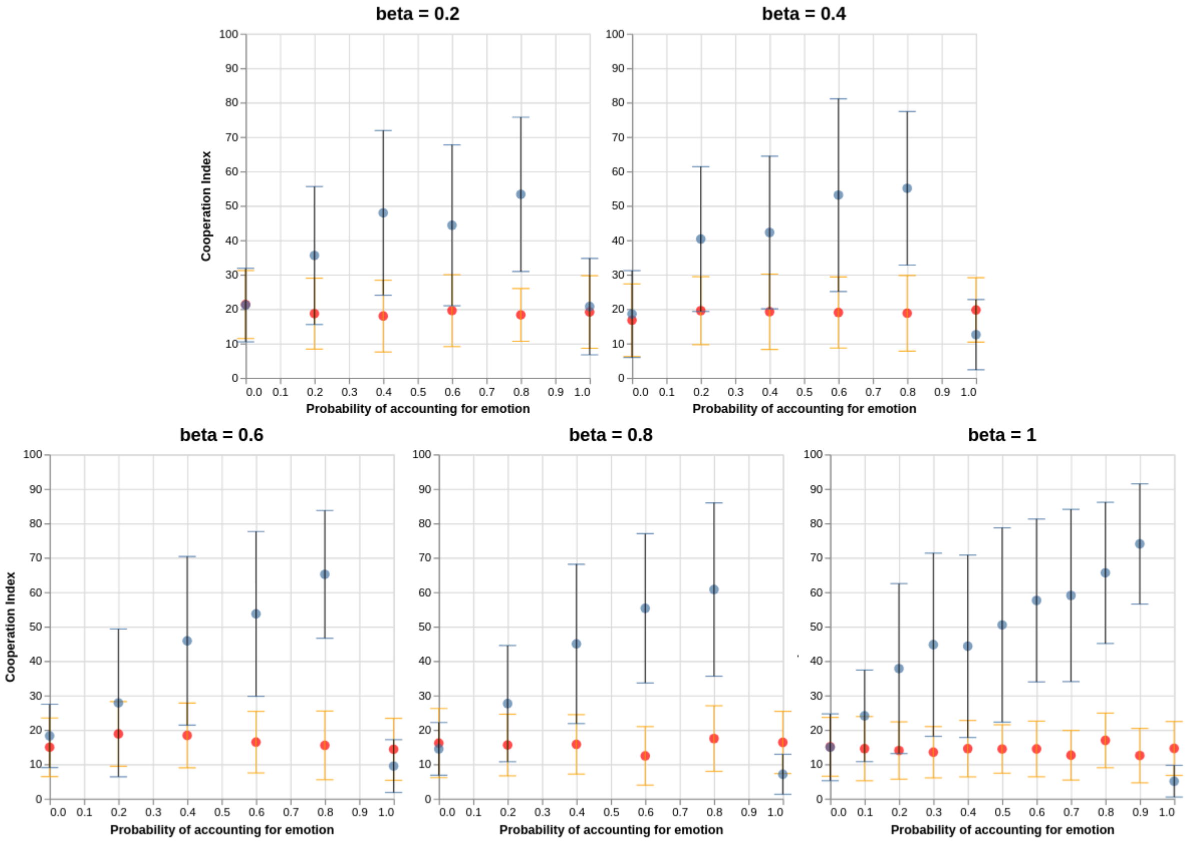

Fig. S6. Analysis of the robustness of cooperation promoted by EBIS to different values of selection intensity. The main result – using emotion expressions generally leads to higher cooperation indexes – remains true for all studied selection intensity values.

SI References

(Brandt and Sigmund, 2006) *Brandt, H., & Sigmund, K. (2006). The good, the bad and the discriminator—Errors in direct and indirect reciprocity. Journal of Theoretical Biology, 239(2), 183–194.* https://doi.org/10.1016/J.JTBI.2005.08.045

(Fishman, 2003) *Fishman, M. A. (2003). Indirect reciprocity among imperfect individuals. Journal of Theoretical Biology, 225(3), 285–292.* https://doi.org/10.1016/S0022-5193(03)00246-7

(Sommerfeld et al, 2007) *Sommerfeld, R. D., Krambeck, H. J., Semmann, D., & Milinski, M. (2007). Gossip as an alternative for direct observation in games of indirect reciprocity. Proceedings of the National Academy of Sciences of the United States of America, 104(44), 17435–17440.* https://doi.org/10.1073/pnas.0704598104

(Ohtsuki and Iwasa, 2004*) Ohtsuki, H., & Iwasa, Y. (2004). How should we define goodness? - Reputation dynamics in indirect reciprocity. Journal of Theoretical Biology, 231(1), 107–120.* https://doi.org/10.1016/j.jtbi.2004.06.005

(Santos et al, 2016a) *Santos, F. P., Pacheco, J. M., & Santos, F. C. (2016). Evolution of cooperation under indirect reciprocity and arbitrary exploration rates. Scientific Reports, 6(August), 1–9.* https://doi.org/10.1038/srep37517

(Traulsen et al, 2006) *Traulsen, A., Nowak, M. A., & Pacheco, J. M. (2006). Stochastic dynamics of invasion and fixation. Physical Review E - Statistical, Nonlinear, and Soft Matter Physics, 74(1), 011909.* https://doi.org/10.1103/PHYSREVE.74.011909/FIGURES/1/MEDIUM

(Nowak and Sigmund, 1998) *Nowak, M. A., & Sigmund, K. (1998). Evolution of indirect reciprocity by image scoring. Nature, 393(6685), 573–577.* https://doi.org/10.1038/31225

(de Melo et al, 2021) *de Melo, C. M., Terada, K., & Santos, F. C. (2021). Emotion expressions shape human social norms and reputations. IScience, 24(3), 102141.* https://doi.org/10.1016/j.isci.2021.102141

(Pacheco et al, 2006) *Pacheco, J. M., Santos, F. C., & Chalub, F. A. C. C. (2006). Stern-judging: A simple, successful norm which promotes cooperation under indirect reciprocity. PloS Computational Biology, 2(12), 1634–1638.* https://doi.org/10.1371/journal.pcbi.0020178

(Santos et al, 2016b) *Santos, F. P., Santos, F. C., & Pacheco, J. M. (2016). Social Norms of Cooperation in Small-Scale Societies. PLoS Computational Biology, 12(1), 1–13.* https://doi.org/10.1371/journal.pcbi.1004709

(Uchida, 2010) *Uchida, S. (2010). Effect of private information on indirect reciprocity. Physical Review E - Statistical, Nonlinear, and Soft Matter Physics, 82(3), 1–8.* https://doi.org/10.1103/PhysRevE.82.036111

(Hilbe et al, 2018) *Hilbe, C., Schmid, L., Tkadlec, J., Chatterjee, K., & Nowak, M. A. (2018). Indirect reciprocity with private, noisy, and incomplete information. Proceedings of the National Academy of Sciences of the United States of America, 115(48), 12241–12246.* https://doi.org/10.1073/pnas.1810565115
